# Implant placement accuracy using computer-guided surgical templates supported by bone and mucosa: an *in vitro* study

**DOI:** 10.1590/1678-7765-2025-0926

**Published:** 2026-07-27

**Authors:** Ana Maria Alves Ribeiro, Déborah Rocha Seixas, Eduardo Sanches Gonçales

**Affiliations:** 1 Universidade de São Paulo Faculdade de Odontologia de Bauru Departamento de Cirurgia Bauru SP Brasil Universidade de São Paulo, Faculdade de Odontologia de Bauru, Departamento de Cirurgia, Estomatologia, Patologia e Radiologia, Bauru, SP, Brasil.

**Keywords:** Dental Implants, Surgery, Computer-Assisted, *In vitro* techniques, Surgery,oral

## Abstract

Computer-guided surgery, which combines tomographic data and digital models, has been widely used in implant dentistry to improve implant placement accuracy.

**Objective:**

This *in vitro* study evaluated the accuracy of virtual planning for single implant placement in the regions of teeth 33 and 43, comparing bone-supported and mucosa-supported guides. A secondary analysis assessed differences related to insertion method, since implant placement in the 43 region used a manual ratchet and in the 33 region used a motorized handpiece.

**Methodology:**

In total, 32 implants were placed in 16 edentulous artificial mandibles (8 using bone-supported guides and 8 using mucosa-supported guides), with two implants per mandible. Implant positions were assessed using computed tomography and compared with the virtual plan using ImageJ (2.16.0/1.54p) and OneViewerApp (version 1.18.0.0, 2023). Statistical analysis was performed on Jamovi (*p*<0.05). Linear deviations between the planned and actual implant placement were compared using t-test or the Mann-Whitney U test, depending on data distribution.

**Results:**

Results indicated greater deviations for mucosa-supported guides, especially in the buccolingual direction, whereas bone-supported guides exhibited greater accuracy.

**Conclusion:**

Within the limitations of this *in vitro* study, the type of guide support may influence implant placement accuracy and should be carefully considered, particularly in procedures requiring high precision. Further studies are needed to confirm these findings in clinical settings.

## INTRODUCTION

Computer-guided surgery has been widely adopted in implant dentistry due to its ability to improve the accuracy and predictability of dental implant placement. This approach involves combining tomographic images and digital models for virtual planning, followed by the fabrication of surgical guides which can be tooth-, bone-, or mucosa-supported.^1^ Tooth-supported guides generally provide high positional accuracy due to their rigid stabilization; however, their use is limited to partially edentulous patients.^2^ In edentulous cases, surgical guidance relies exclusively on bone- or mucosa-supported designs, both of which present specific limitations. Bone-supported guides require flap elevation, increasing surgical invasiveness and complexity.^3^ In turn, mucosa-supported guides depend on the thickness and resilience of the soft tissue, which may allow for displacement during drilling and implant insertion.^4^

Recent systematic reviews and experimental studies have reported varying deviations between planned and placed implants in edentulous conditions, particularly in the buccolingual direction, along with clinically relevant angular discrepancies.^3,5^ However, many comparative studies, such as that by Markiewicz and Nowicki^6^ (2025), include tooth-supported guides in their analyses which may overestimate the overall accuracy of guided surgery and limit extrapolation to edentulous scenarios. Consequently, evidence regarding the performance of guided placement in edentulous mandibles remains inconsistent.^7^ These deviations in implant positioning can result in significant complications.^8^ Deviations in angulation or depth during placement may compromise prosthetic outcomes and increase the risk of biological and mechanical complications,^9^ whereas improper placement increases the risk of damage to adjacent vital anatomical structures.^8,10^ Thus, identifying the most stable support design for edentulous arches remains essential for improving implant placement accuracy and surgical predictability.

The primary objective of this *in vitro* study was to compare implant placement accuracy using bone-supported and mucosa-supported guides exclusively in edentulous artificial mandibles. As a secondary objective, we evaluated the influence of the implant insertion method (manual ratchet versus motorized handpiece) on the final implant position. By isolating these variables in a controlled experimental model, this study seeks to clarify the biomechanical factors that affect the accuracy of guided implants under edentulous conditions.

## METHODOLOGY

This *in vitro* experimental study was designed and reported according to the guidelines established for publishing preclinical laboratory studies in dentistry.^11^ Since the research was conducted exclusively with artificial models, without involvement of human participants, biological specimens, or patient data, ethical approval was not required.

A total of 16 edentulous artificial mandibles (TitaniumFix, São José dos Campos, SP, Brazil; Batch 9023/24) were divided into two groups (n=8 per group): bone-supported guides and mucosa-supported guides (Figure 1). Each mandible received two implants, totaling 32 implants. The implant was considered the unit of analysis. Sample size was determined based on methodological standardization and experimental feasibility, in line with previously published similar *in vitro* studies, including those by Taheri Otaghsara, Joda and Thieringer^12^ (2023). Of the 20 mandibles initially selected (n=10 per group), four were excluded due to technical issues during the digital workflow and fabrication of the surgical guide (FastGuide Fluxo Digital, São Paulo, Brazil). All procedures were performed by a single trained operator to maintain standardization throughout the study. Blinding was not feasible due to the inherent nature of the experimental design.

**Figure 1:**
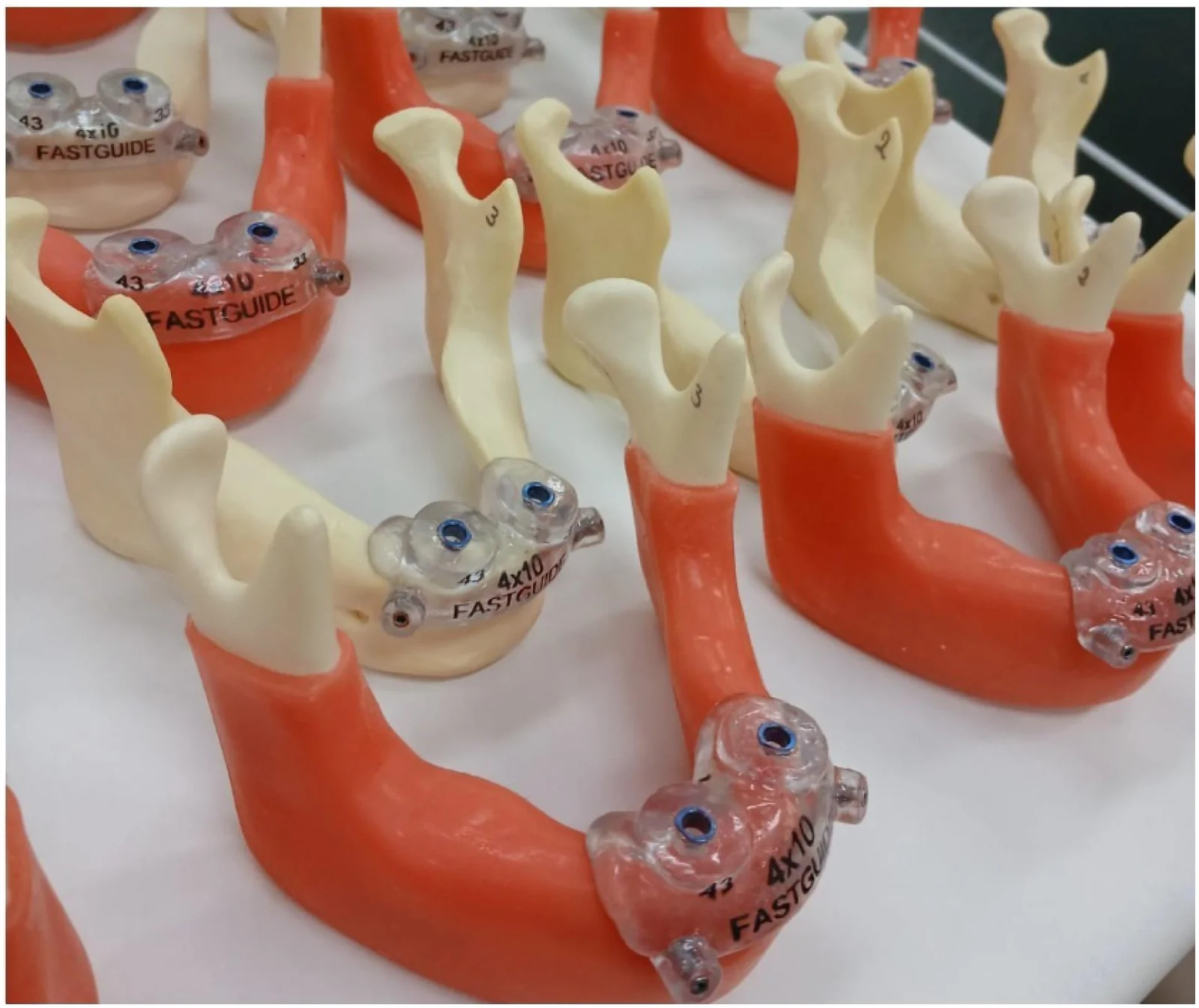
Surgical guides positioned on the edentulous mandibles to assess passive adaptation prior to stabilization with fixation pins.

All mandibles were scanned using a Virtuo Vivo scanner (Straumann, Basel, Switzerland) to obtain STL data. Cone-beam computed tomography (CBCT) images were acquired using an XG3D unit (Planmeca, Helsinki, Finland) with a field of view of 140×50 mm, 70 kV, and 6.0 mA. STL and DICOM datasets were sent to Fastguide Fluxo Digital (São Paulo, Brazil) for fabrication of the surgical guides (Figure 1). Regions 33 and 43 were selected as implant sites because they correspond to anatomically comparable positions in the mandibular canine region, allowing for standardized conditions in fully edentulous models. From a clinical perspective, implant placement in edentulous mandibles is commonly performed in the interforaminal region to support prosthetic rehabilitation, making these sites both relevant and suitable for experimental comparison.

A fully guided surgical system (Guide-fix, TitaniumFix) was used, along with Profile 4.0 × 10.0 mm Black Fix self-tapping implants (TitaniumFix, São José dos Campos, SP, Brazil; Batch 1728/24). Two implants were placed per mandible, in accordance with the virtual planning. The surgical guide remained fixed during drilling and implant insertion. All mandibles were stabilized on a workbench to ensure standardization. In region 43, the implants were inserted using a manual ratchet; in region 33, insertion was performed using a motorized handpiece.

Implant positions were evaluated by comparing the preoperative virtual planning with the postoperative CBCT scans using ImageJ (U.S. National Institutes of Health, Bethesda, MD, USA) and OneViewerApp (OneDigital, São Paulo, Brazil). Sagittal and lateral deviations were calculated based on predefined reference points. For lateral measurements, a central reference line was established between the implants, and measurements were taken 20 mm on each side to determine mesial and distal deviations. For sagittal measurements, buccolingual deviations were assessed from the central region of each implant to the limit of the surrounding structure (bone or mucosa). For mucosa-supported mandibles, the mucosal thickness was standardized at 3 mm across all samples and confirmed via CBCT and clinical inspection.

Deviation variables were classified into six sagittal parameters (buccal: IV1, IV2, IV3; lingual: IL1, IL2, IL3) and six lateral parameters (mesial: M1, M2, M3; distal: D1, D2, D3). The numerical designation corresponded to coronal implant levels: 1 (implant head/cervical), 2 (middle), and 3 (apex) (Figure 2).

**Figure 2:**
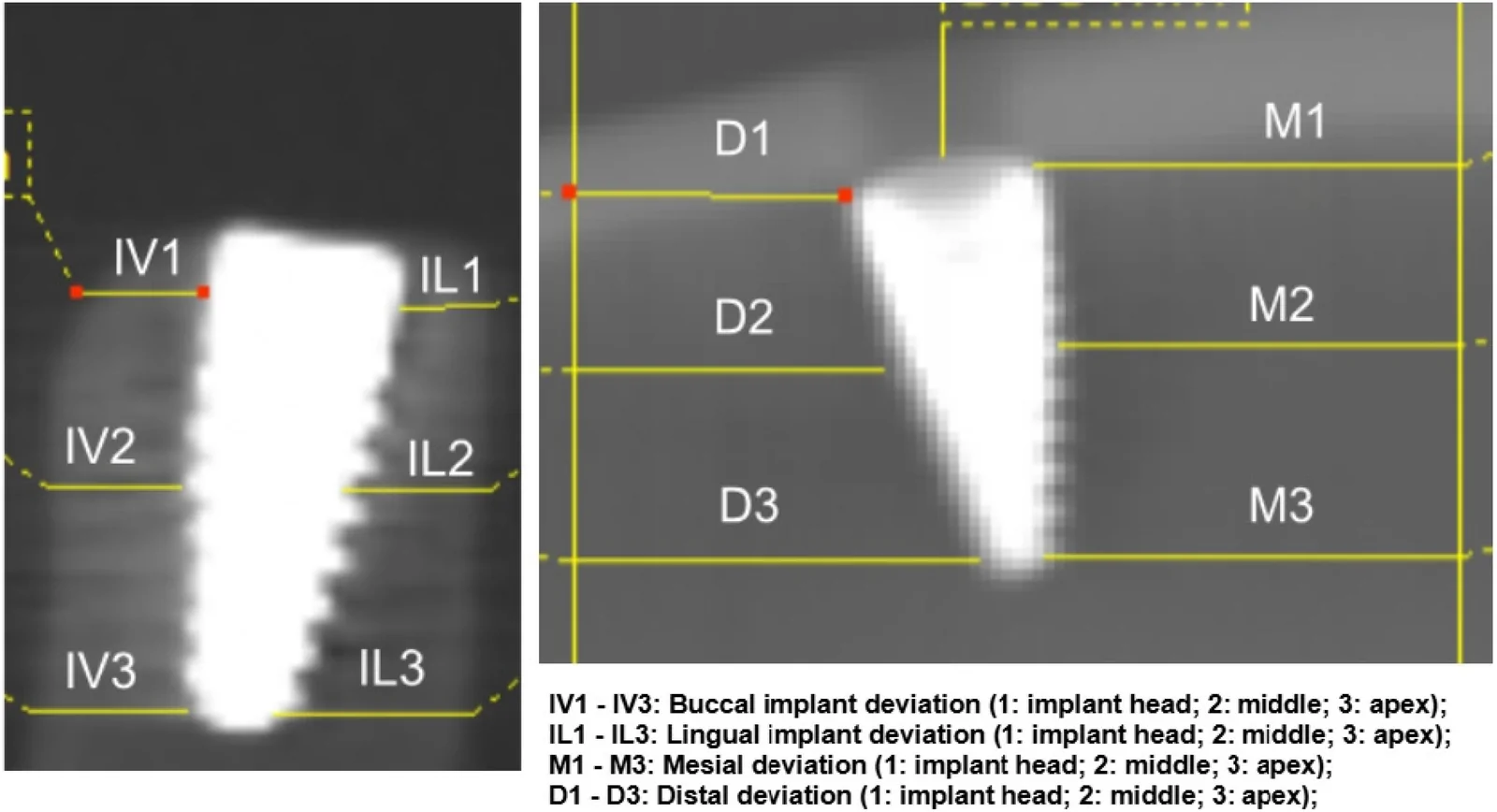
Selected variables and occlusal reference line determining the 3-mm distance from the mucosal surface.

Statistical analysis to compare the lateral and sagittal deviations between planned and actual implant placement was performed on Jamovi software (version 2.3.16, 2023). When the assumptions of normality were met, parametric tests were applied; otherwise, corresponding non-parametric alternatives were used. Intergroup analysis applied the independent-samples *t*-test or the Mann–Whitney U test, as appropriate. Intragroup comparisons used the *t*-test for paired-samples or the Wilcoxon signed-rank test. Significance level was set at 5% (*p*<0.05).

## RESULTS

The intergroup descriptive analysis (Table 1), which compared differences between the mucosa- and bone-supported guide groups, revealed higher mean values and standard deviations in the mucosa-supported guide group, particularly for the buccolingual parameters, compared with the bone-supported guide group.

**Table 1:** Descriptive statistics of deviations for tooth 33 and tooth 43, sequentially.

		Tooth 33 - handpiece	Tooth 43 - manual ratchet
		(Mean ± SD)	(Mean ± SD)
IV1DIF (mm)	Mucosa	0.931±0.776	0.765±0.611
	Bone	0.510±0.218	0.517±0.423
IV2DIF (mm)	Mucosa	1.071±0.838	0.759±0.562
	Bone	0.224±0.160	0.845±0.534
IV3DIF (mm)	Mucosa	1.198±0.937	0.739±0.618
	Bone	0.047±0.340	0.336±0.275
IL1DIF (mm)	Mucosa	0.945±0.986	0.755±0.557
	Bone	0.563±0.393	0.733±0.418
IL2DIF (mm)	Mucosa	1.377±1.217	1.300±0.781
	Bone	0.408±0.244	0.629±0.458
IL3DIF (mm)	Mucosa	2.123±1.652	1.665±0.863
	Bone	0.411±0.222	0.454±0.574
M1DIF (mm)	Mucosa	0.961±0.902	2.755±2.447
	Bone	1.716±0.872	1.185±1.298
M2DIF (mm)	Mucosa	1.278±1.156	2.913±2.226
	Bone	1.491±0.982	1.330±1.083
M3DIF (mm)	Mucosa	1.414±1.747	3.004±2.179
	Bone	1.699±0.881	1.495±1.454
D1DIF (mm)	Mucosa	1.036±0.532	2.072±1.334
	Bone	2.395±1.481	2.158±1.779
D2DIF (mm)	Mucosa	1.144±0.905	2.524±1.335
	Bone	2.559±1.486	2.192±2.480
D3DIF (mm)	Mucosa	1.321±1.233	3.020±2.278
	Bone	2.151±1.281	2.049±2.213

IV1 - IV3: Buccal implant deviation (1:implant head; 2: middle; 3: apex)

IL1 - IL3: Lingual implant deviation (1:implant head; 2: middle; 3: apex);

M1 - M3: Mesial deviation (1:implant head; 2: middle; 3: apex);

D1 - D3: Distal deviation (1:implant head; 2: middle; 3: apex);

DIF: Difference between virtual planning and postoperative outcome;

SD: Standard deviation

Regarding mucosa-supported guides (Table 1), tooth 33 showed significant differences between planned and postoperative measurements for IV1 (*p*=0.023), IV2 (*p*=0.047), IV3 (*p*=0.019), IL2 (*p*=0.027), and IL3 (*p*=0.015). For tooth 43, significant differences were observed for IL1 (*p*=0.017), IL2 (*p*=0.004), IL3 (*p*<0.001), M2 (*p*=0.041), and M3 (*p*=0.035).

Conversely, the significant differences in bone-supported guides were limited to a single variable per dental region. For tooth 33, significant difference was observed only for IV3 (*p*=0.006), whereas for tooth 43 the significant difference occurred in IL3 (*p*=0.022).

Intergroup analysis, which compared the differences between planned and actual variables in mucosa-supported versus bone-supported mandibles, found significant differences particularly for tooth 33, including IV2 (p=0.014), IL2 (p=0.044), IL3 (p=0.012), D1 (p=0.028), and D2 (p=0.037) (Table 1). For tooth 43, only IL3 showed a significant difference between the types of surgical guides (p=0.005) (Table 1).

## DISCUSSION

Results revealed significant deviations between virtual planning and postoperative outcomes, with a higher prevalence in mucosa-supported guides, particularly in the buccolingual direction. These deviations are likely related to the thickness and elasticity of the mucosa which affect the stability and precise fit of the surgical guide. Previous studies have reported reduced accuracy with mucosa-supported guides due to limited tissue support, especially in cases of increased mucosal thickness or mobility, thereby increasing the risk of linear and angular deviations.^3,13^

Additionally, the insertion technique appeared to have influenced the results. The implant placed in region 43 using a manual ratchet showed greater accuracy than the implant in region 33, inserted with a motorized handpiece. Interpreting this observation requires caution, however, as it may be influenced not only by the insertion method but also by factors such as implant location, initial implant stability, surgical time, and insertion torque.^14^ Although most studies do not report statistically significant differences between insertion techniques in guided surgeries, the present findings revealed a discrepancy between the evaluated approaches.^14^ Notably, our comparison between insertion techniques involved different implant sites (teeth 43 and 33), which may have introduced confounding factors related to anatomical or positional aspects. Despite both sites corresponding to anatomically similar regions (mandibular canines) and using a standardized *in vitro* model, this design does not allow complete control of potential confounding variables. Thus, the observed differences cannot be attributed exclusively to the insertion technique and should be interpreted with caution.

Previous clinical studies have shown that guided implant surgery accuracy can be influenced by variables related to surgical protocol and technical workflow.^9,15^ Deviations tend to be greater in the buccolingual than in the mesiodistal direction, likely due to differences in structural support and tissue behavior. In the mesiodistal direction, adjacent teeth or mandibular morphology can help stabilize the guide, limiting lateral displacement. In the buccolingual direction, however, especially with mucosa-supported guides, the compressibility of soft tissue may allow for displacement during drilling or implant insertion.^3^ Moreover, guide positioning and fixation, type of guide (full and partial), surgical approach, bone density, mandibular anatomy, and inclination of the alveolar crest are well-known factors contributing to angular and linear deviations.^2^

Despite the significance of these findings, certain limitations must be acknowledged. Since we used an artificial model, clinical conditions such as involuntary patient movement and tissue variability were not replicated. Operator-related factors and potential implant wear may also have affected the results. Previous studies similarly indicate that surgeon experience and the type of guide support (mucosal or bone) significantly influence guided surgery accuracy.^16,17^ Another limitation is the potential measurement variability associated with using different software tools. Our study employed two applications, which may have introduced minor discrepancies due to differences in processing algorithms and interpretation of measurements. Previous research indicates that software selection can influence data reproducibility, particularly in three-dimensional analysis based on computed tomography.^5,18^ Moreover, the present analysis was limited to linear deviations, whereas other studies on guided implant surgery also report angular and overall three-dimensional ones. This may restrict direct comparison with the literature and limit the overall comprehensiveness of the accuracy assessment. Thus, future studies should consider standardized measurement protocols to reduce potential biases.

Moreover, the study includes a limited number of samples (n=32) and no formal statistical power calculation was performed. While this may be acceptable for an exploratory *in vitro* study, it limits the robustness of the conclusions and should be considered when interpreting the findings. Exclusion of some samples due to technical issues represents an additional limitation, as it may have introduced bias or influenced the overall representativeness of the sample. Notably, these exclusions referred to difficulties in accurately superimposing STL and DICOM data during the digital workflow, which prevented proper guide fabrication. This issue may be associated with inaccuracies in image acquisition or data processing steps (e.g., CBCT or surface scanning), and highlights a potential limitation of the digital workflow in guided surgery. Such factors should therefore be considered both in the interpretation of the present results and in the planning of clinical applications.

These findings underscore the importance of optimizing the fit of surgical guides to improve the predictability of computer-assisted implant surgery, particularly when using mucosa-supported guides. Tissue characteristics and guide stability appear to directly influence accuracy and should be carefully considered during treatment planning. Further research is needed to refine guide design and clinical protocols, thereby enhancing the reliability of these systems across diverse clinical settings.

## CONCLUSION

Within the limitations of this *in vitro* study, bone-supported guides were associated with greater accuracy in implant placement compared with mucosa-supported guides. Thus, the elasticity and thickness of the mucosa should be considered when selecting the type of guide. Moreover, the implant insertion method may influence accuracy relative to virtual planning, suggesting that it may play a role in clinical outcomes and should be considered during implant placement. Further studies are needed to confirm these observations under clinical conditions.

## Data Availability

All data generated or analyzed during this study are included in this published article.

## References

[1] Bover-Ramos F, Viña-Almunia J, Cervera-Ballester J, Peñarrocha-Diago M, García-Mira B (2018). Accuracy of implant placement with computer-guided surgery: a systematic review and meta-analysis comparing cadaver, clinical, and in vitro studies. Int J Oral Maxillofac Implants.

[2] Azevedo M, Correia F, Faria Almeida R (2024). Accuracy of implant guided surgery in fully edentulous patients: prediction vs. actual outcome-systematic review. J Clin Med.

[3] Seo C, Juodzbalys G (2018). Accuracy of guided surgery via stereolithographic mucosa-supported surgical guide in implant surgery for edentulous patient: a systematic review. J Oral Maxillofac Res.

[4] Deeb JG, Bencharit S, Loschiavo CA, Yeung M, Laskin D, Deeb GR (2018). Do implant surgical guides allow an adequate zone of keratinized tissue for flapless surgery?. J Oral Maxillofac Surg.

[5] Zhou W, Feng G, Luo Z, Xu L, Cao Y, Song K (2025). Comparison of the accuracy of guided implant surgery between two implant-planning software: a retrospective cohort study. Front Oral Health.

[6] Markiewicz M, Nowicki AA (2025). Accuracy of computer-guided dental implant placement: a clinical comparison of three surgical guide types. J Clin Med.

[7] Raico Gallardo YN, Silva-Olivio IR, Mukai E, Morimoto S, Sesma N, Cordaro L (2017). Accuracy comparison of guided surgery for dental implants according to the tissue of support: a systematic review and meta-analysis. Clin Oral Implants Res.

[8] Alaqeely R, Albaiz A, Alenazi B, Alem M, Alotaibi Y, Alrowis R (2025). Prevalence of dental implant positioning errors: a radiographic analysis. J Clin Med.

[9] Sadilina S, Vietor K, Doliveux R, Siu A, Chen Z, Al-Nawas B (2025). Beyond accuracy: clinical outcomes of computer assisted implant surgery. Clin Exp Dent Res.

[10] Ma B, Park T, Chun I, Yun K (2018). The accuracy of a 3D printing surgical guide determined by CBCT and model analysis. J Adv Prosthodont.

[11] Krithikadatta J, Gopikrishna V, Datta M (2014). CRIS guidelines (Checklist for Reporting In-vitro Studies): a concept note on the need for standardized guidelines for improving quality and transparency in reporting in-vitro studies in experimental dental research. J Conserv Dent.

[12] Taheri Otaghsara SS, Joda T, Thieringer FM (2023). Accuracy of dental implant placement using static versus dynamic computer-assisted implant surgery: an in vitro study. J Dent.

[13] D'haese R, Vrombaut T, Hommez G, Bruyn HD, Vandeweghe S (2021). Accuracy of guided implant surgery in the edentulous jaw using desktop 3D-printed mucosal supported guides. J Clin Med.

[14] Orban K, Varga E, Windisch P, Braunitzer G, Molnar B (2022). Accuracy of half-guided implant placement with machine-driven or manual insertion: a prospective, randomized clinical study. Clin Oral Investig.

[15] Marquez Bautista N, Meniz-García C, López-Carriches C, Sánchez-Labrador L, Cortés-Bretón Brinkmann J, Madrigal Martínez-Pereda C (2024). Accuracy of different systems of guided implant surgery and methods for quantification: a systematic review. Appl Sci (Basel).

[16] Shi Y, Wang J, Ma C, Shen J, Dong X, Lin D (2023). A systematic review of the accuracy of digital surgical guides for dental implantation. Int J Implant Dent.

[17] Schulz MC, Rittmann L, Range U, Lauer G, Haim D (2018). The use of orientation templates and free-hand implant insertion in artificial mandibles: an experimental laboratory examination in fifth-year dental students. Dent J (Basel).

[18] Bjelica R, Smojver I, Stojić L, Vuletić M, Katanec T, Gabrić D (2026). Accuracy of fully guided implant placement using bone-supported stackable surgical guides in completely edentulous patients: a retrospective study. J Clin Med.

